# Hypercholesterolemia and apolipoprotein B expression: Regulation by selenium status

**DOI:** 10.1186/1476-511X-4-28

**Published:** 2005-11-05

**Authors:** Sanjiv Dhingra, Mohinder P Bansal

**Affiliations:** 1 Department of Biophysics, Panjab University, Chandigarh-160014, India

## Abstract

**Background:**

Apolipoprotein B (apoB) contains ligand-binding domain for the binding of LDL to LDL-R site, which enables the removal of LDL from circulation. Our recent data showed that selenium (Se) is involved in the lipid metabolism. The present study was aimed to understand the effect of Se deficiency (0.02 ppm) and selenium supplementation (1 ppm) on apoB expression in liver during hypercholesterolemia in male Sprague Dawley rats. Animals were fed with control and high cholesterol diet (2%) for 1 and 2 months. ApoB levels by ELISA and protein expression by western blot was done. Hepatic LDL receptor (LDL-R) activity (in vivo) and mRNA expression by RT-PCR was monitored.

**Results:**

In selenium deficiency and on high cholesterol diet (HCD) feeding apoB levels increased and LDL-R expression decreased significantly after 2 months. On 1 ppm selenium supplementation apoB expression significantly decreased and LDL-R expression increased after 2 months. But after one month of treatment there was no significant change observed in apoB and LDL-R expression.

**Conclusion:**

So the present study demonstrates that Se deficiency leads to up regulation of apoB expression during experimental hypercholesterolemia. Selenium supplementation upto 1 ppm leads to downregulation of apoB expression. Further, this study will highlight the nutritional value of Se supplementation in lipid metabolism.

## Background

The interaction between LDL and LDL receptor has a major role in determining plasma cholesterol levels [1,2] and apolipoprotein B (apoB) has the central role in this ligand-receptor interaction. Mutations in the apoB gene result in accumulation of LDL in circulation [3,4]. However, most of the studies suggested that one molecule of apoB exists per lipoproteinparticle, thus the quantity of apoB in fasting plasma predicts the number of LDL and VLDL particles [5,6]. Therefore, plasma apoB levels maybe a better assay of the concentration of atherogenic lipoproteinparticles than total or LDL cholesterol levels [7]. Furthermore, a cross-sectional study in patients who had coronary arterybypass graft surgery determined that apoB concentration wasa better discriminator than LDL cholesterol concentration inpredicting recurrent atherosclerotic disease in bypass grafts10 years after surgery [8].

Abnormalities in the apoB metabolism are responsible for the generation of hypercholesterolemia and increased risk of coronary heart disease [9]. Homma [10] reported a positive correlation between serum apoB levels and atherosclerotic conditions. Abraham et al. [11] have found a significantly increased production of apoB levels in cultured hepatocytes isolated from rats fed with atherogenic diet. Incubation of hepatocytes isolated from normal rats with added cholesterol resulted in an increased synthesis and secretion of apoB levels [12].

Several studies suggested that T_3 _is directly involved in the regulation of LDL-R and apoB expression [13,14]. Normally thyroid is the unique source of T_4_, but it secretes only 20% of the whole T_3 _in the body. Major amount of T_3 _is produced from T_4 _by 5'-deiodination in peripheral tissues [15]. This reaction is catalyzed by type-I 5'-iodothyronine deiodinase (5'-DI). Type-1 5'-iodothyronine deiodinase being a selenoprotein its activity decreases during selenium (Se) deficiency [16,17]. Hence during Se deficiency, T_3 _can not be produced in any quantity. This makes the role of Se important for lipid metabolism, since T_3 _is known to regulate the LDL-R level, which is further responsible for the maintenance of plasma cholesterol level. Wojcicki et al. [18] reported the protective role of Se against atherosclerosis.

Hence in view of all the above stated findings, present study is aimed to understand the effect of Se status on apoB levels under experimental hypercholesterolemic conditions in SD male rats. To the best of our knowledge, so far no other study has been reported linking Se status with apoB expression during hypercholesterolemia.

## Results

### Selenium levels

Se levels in the serum and liver decreased significantly (p < 0.001) in Se deficient groups (Ia and Ib) and increased in 1 ppm Se supplemented diet fed groups (IIIa and IIIb) in comparison to respective adequate groups (IIa and IIb). Significant decrease (p < 0.001) in the level was observed in HCD fed groups as compared to respective controls in all the three Se status i.e. deficient, adequate and excess groups. In deficient groups (Ia and Ib) and in HCD fed adequate group, Se level decreased significantly (p < 0.001), whereas in 1 ppm Se supplemented groups the level increased significantly (p < 0.001) after 2 months in comparison to 1-month data (Table 1).

**Table 1:** Selenium levels in liver (μg/g) and serum (μg/L), GSH-Px levels in liver (μmoles of NADPH oxidized/min/mg protein) after 1 and 2 months of control and high cholesterol diet (HCD) feeding.

**Treatment period**	**Se deficient group**		**Se adequate group**		**Se excess group**	
	**Control Ia**	**HCD Ib**	**Control IIa**	**HCD IIb**	**Control IIIa**	**HCD IIIb**

**Se in liver**						
1 month	1.83 ± 0.26^DDD^	1.20 ± 0.14^***###^	3.85 ± 0.29	3.11 ± 0.39**	4.57 ± 0.43^LL^	2.51 ± 0.29^***B^
2 months	1.23 ± 0.22^DDDAA^	0.73 ± 0.05^***AAA###^	3.97 ± 0.36	2.54 ± 0.27^***A^	6.51 ± 0.65^AAALLL^	4.38 ± 0.44^***AAABBB^
Se in serum						
1 month	1.54 ± 0.15^DDD^	0.76 ± 0.04^***###^	2.51 ± 0.24	2.11 ± 0.27*	3.84 ± 0.34^LLL^	2.62 ± 0.27^***BB^
2 month	0.98 ± 0.07^DDDAAA^	0.51 ± 0.02^***AAA###^	2.82 ± 0.29	1.62 ± 0.17^***AA^	4.73 ± 0.39^AALLL^	3.58 ± 0.31^***AAABBB^
**GSH-Px**						
1 month	281.45 ± 34.89^DDD^	354.19 ± 32.79^**###^	387.42 ± 25.86	467.19 ± 27.66***	568.82 ± 52.94^LLL^	686.19 ± 50.58^**BBB^
2 month	160.33 ± 22.78^DDDAAA^	463.73 ± 39.91^***AAA###^	393.16 ± 49.67	593.28 ± 35.38^***AAA^	672.91 ± 58.16^AALLL^	825.16 ± 63.85^**AABBB^

Data is represented as mean ± SD from 6 observations.

*p<0.05, **p<0.01, ***p<0.001 represent comparison between control and HCD groups; ^A^*p*<0.05, ^AA^*p*<0.01, ^AAA^*p*<0.001 comparison between 1 and 2 months; ^DDD^*p*<0.001 comparison between group Ia and IIa; ^###^*p*<0.001 comparison between group Ib and IIb; ^LL^*p*<0.01, ^LLL^*p*<0.001 comparison between group IIa and IIIa; ^B^*p*<0.05, ^BB^*p*<0.01, ^BBB^*p*<0.001 comparison between group IIb and IIIb.

### Glutathione peroxidase activity

Glutathione peroxidase (GSH-Px) activity in liver decreased significantly (p < 0.001) in Se deficiency (Ia and Ib) and it increased on 1 ppm Se supplementation (IIIa and IIIb) in comparison to respective adequate groups (IIa and IIb). On HCD feeding significant increase (p < 0.001) was observed in all the groups in comparison to respective controls. In Se deficient control group GSH-Px level decreased, whereas in HCD supplemented Se deficient and Se adequate as well as excess Se fed groups the level increased significantly (p < 0.001) after 2 months as compared to 1-month data (Table 1).

### Total Cholesterol and LDL Level

In all the three Se status groups on HCD feeding significant increase (p < 0.001) in total cholesterol and LDL-cholesterol concentration was observed in comparison to respective control groups. In Se deficient groups (Ia and Ib) total cholesterol and LDL level increased and on 1 ppm Se supplementation it decreased significantly (p < 0.001) in comparison to respective adequate groups (IIa and IIb). In both the Se deficient groups (Ia and Ib) and in HCD fed adequate group lipid level increased significantly (p < 0.001), whereas it decreased on Se supplementation after 2 months in comparison to 1-month treatment period (Table 2).

**Table 2:** Total cholesterol (mg/dl), LDL-cholesterol (mg/dl) and apolipoprotein B (A_405_) levels in serum after 1 and 2 months of control and high cholesterol diet (HCD) feeding schedule.

**Treatment period**	**Se deficient group**		**Se adequate group**		**Se excess group**	
	**Control Ia**	**HCD Ib**	**Control IIa**	**HCD IIb**	**Control IIIa**	**HCD IIIb**

**Cholesterol**						
1 month	107.87 ± 6.87^DDD^	246.54 ± 11.96^##***^	83.62 ± 5.45	215.23 ± 13.45***	74.43 ± 6.28^L^	184.29 ± 11.73^***BB^
2 months	120.23 ± 9.53^DDDA^	295.14 ± 13.43^##***AAA^	85.23 ± 5.24	269.82 ± 10.67^***AAA^	62.73 ± 5.38^AALLL^	165.34 ± 10.60^***BBBA^
**LDL**						
1 month	41.47 ± 4.23^DDD^	94.82 ± 7.38^#***^	32.16 ± 2.29	82.78 ± 7.17***	28.62 ± 2.42^L^	70.88 ± 5.36^***BB^
2 months	48.35 ± 3.71^ADDD^	113.50 ± 9.16^#***A^	32.78 ± 2.75	99.73 ± 9.32^***A^	24.12 ± 2.06^AALLL^	60.59 ± 5.83^***BBBAA^
**ApoB**						
1 month	0.195 ± 0.018	0.198 ± 0.019	0.191 ± 0.017	0.196 ± 0.016	0.189 ± 0.016	0.193 ± 0.018
2 months	0.287 ± 0.024^DDDAAA^	0.345 ± 0.028^**AAA##^	0.196 ± 0.016	0.275 ± 0.023^***AAA^	0.130 ± 0.011^AAALLL^	0.162 ± 0.015^**AABBB^

Data is represented as mean ± SD from 6 observations.

**p<0.01, ***p<0.001 represent comparison between control and HCD groups; ^A^*p*<0.05, ^AA^*p*<0.01, ^AAA^*p*<0.001 comparison between 1 and 2 months; ^DD^*p*<0.01, ^DDD^*p*<0.001 comparison between group Ia and IIa; ^##^*p*<0.01, ^###^*p*<0.001 comparison between group Ib and IIb; ^L^*p*<0.05, ^LL^*p*<0.01, ^LLL^*p*<0.001 comparison between group IIa and IIIa; ^BB^*p*<0.01, ^BBB^*p*<0.001 comparison between group IIb and IIIb.

### T_3 _and T_4 _levels

**Figure 1:**
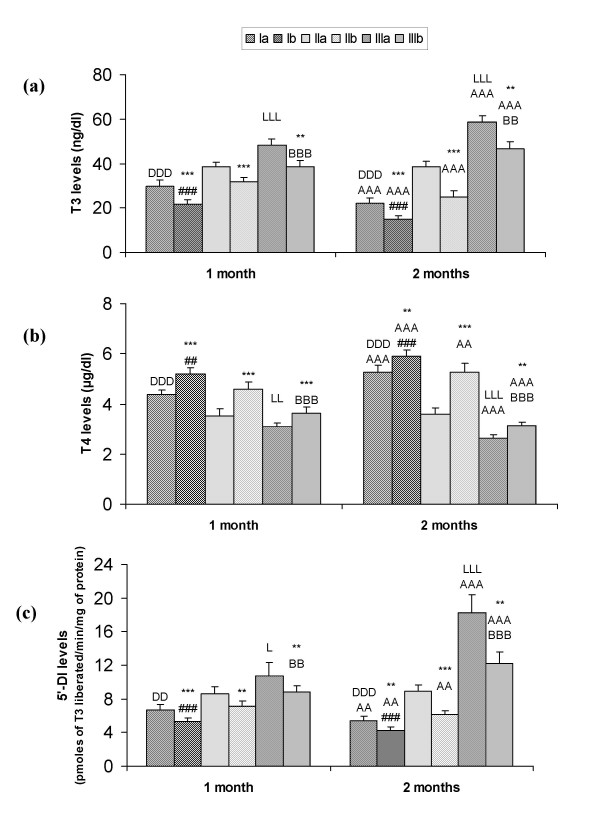
T_3 _(a), T_4 _(b) and 5'-DI (c) levels in serum in different groups: Ia-Se deficient control, Ib-Se deficient+HCD, IIa-Se adequate control, IIb-Se adequate+HCD, IIIa-Se excess control, IIIb-Se excess+HCD after 1 and 2 months. Data is represented as mean ± SD from 6 observations. **p<0.01, ***p<0.001 represent comparison between control and HCD groups; ^AA^*p*<0.01, ^AAA^*p*<0.001 comparison between 1 and 2 months; ^DD^*p*<0.01, ^DDD^*p*<0.001 comparison between group Ia and IIa; ^##^*p*<0.01, ^###^*p*<0.001 comparison between group Ib and IIb; ^L^*p*<0.05, ^LL^*p*<0.01, ^LLL^*p*<0.001 comparison between group IIa and IIIa; ^BB^*p*<0.01, ^BBB^*p*<0.001 comparison between group IIb and IIIb.

Levels of T_3 _decreased and T_4 _increased significantly (p < 0.001) on HCD feeding in comparison to respective controls in all the three Se status groups. In Se deficiency (Ia and Ib) T_3 _decreased and T_4 _level increased in comparison to respective adequate groups, whereas on 1 ppm Se supplementation T_3 _level increased and T_4 _level decreased significantly (p < 0.001). In both the Se deficient groups and in HCD fed adequate group (IIb) T_3 _decreased and T_4 _increased significantly and in Se supplemented groups T_3 _increased and T_4 _decreased after 2 months in comparison to 1-month data (Fig. 1a &1b).

### 5'-DI activity and mRNA expression

**Figure 2:**
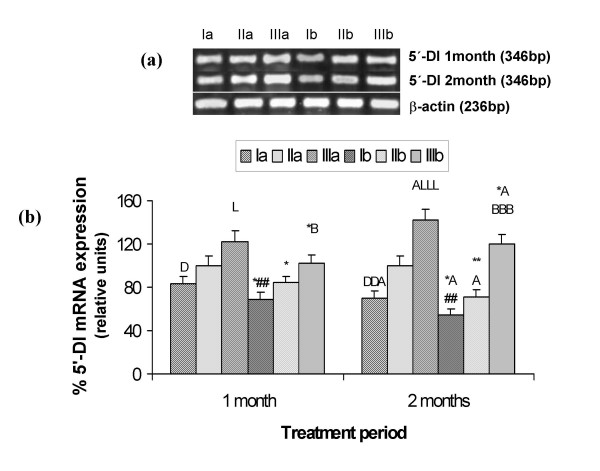
5'-DI mRNA analysis in liver in different groups: Ia-Se deficient control, IIa-Se adequate control, IIIa-Se excess control, Ib-Se deficient+HCD, IIb-Se adequate+HCD, IIIb-Se excess+HCD after 1 and 2 months. (a) mRNA expression by RT-PCR. (b) expression was quantified by densitometric analysis. β-actin was used as an internal control. Data is expressed as mean ± SD from 4 observations. *p<0.05, **p<0.01 represent comparison between control and HCD groups; ^A^*p*<0.05 comparison between 1 and 2 months; ^D^*p*<0.05, ^DD^*p*<0.01 comparison between group Ia and IIa; ^##^*p*<0.01 comparison between group Ib and IIb; ^L^*p*<0.05, ^LLL^*p*<0.001 comparison between group IIa and IIIa; ^B^*p*<0.05, ^BBB^*p*<0.001 comparison between group IIb and IIIb.

5'-DI activity as well as mRNA expression in liver decreased significantly (p < 0.001) on HCD feeding and during Se deficiency in comparison to respective controls. On 1 ppm Se supplementation, significant increase (p < 0.001) in enzyme activity and mRNA expression was observed in comparison to adequate groups. In Se deficient groups (Ia and Ib) and in HCD fed adequate group the activity and mRNA expression decreased and it increased significantly in 1 ppm Se supplemented groups after 2 months in comparison to 1 month data (Fig. 1c and Fig. 2a &2b).

### LDL-R activity and mRNA expression

**Figure 3:**
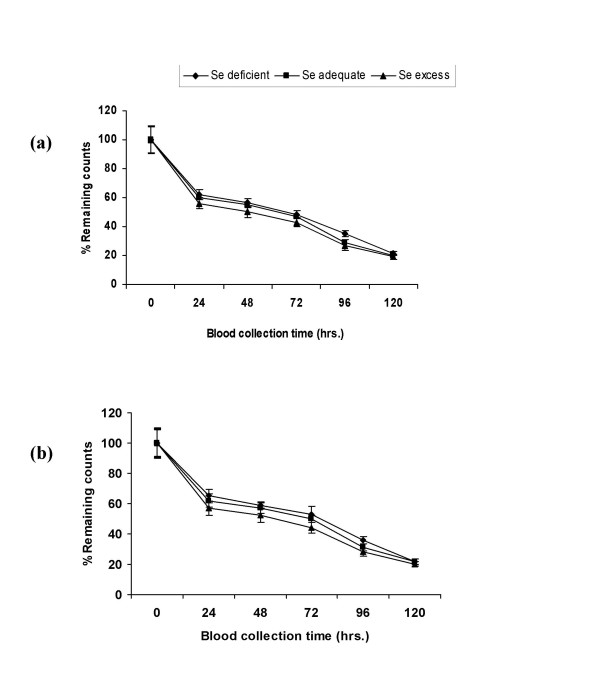
LDL-R activity *in-vivo *during selenium deficiency and on HCD feeding in different groups. (a) after 1 month of control diet feeding. (b) after 1 month of HCD feeding. Radiolabelled LDL was injected to the rats, % decrease in counts in blood with time was taken as a measure of clearance of LDL from animal blood and in turn the LDL-R activity.

**Figure 4:**
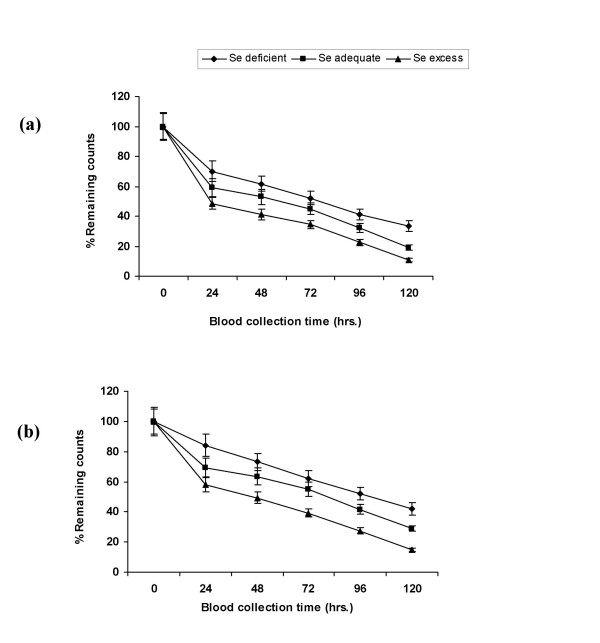
LDL-R activity *in-vivo *during selenium deficiency and on HCD feeding in different groups. (a) after 2 months of control diet feeding. (b) after 2 months of HCD feeding. Radiolabelled LDL was injected to the rats, % decrease in counts in blood with time was taken as a measure of clearance of LDL from animal blood and in turn the LDL-R activity.

Percent remaining counts in blood after 120 h were almost same in all the groups after one month, hence no change was observed in LDL-R activity after one month of diet feeding schedule (Fig. 3a &3b). However after 2 months, the percent remaining counts were higher in Se deficient groups in comparison to adequate diet fed groups, so the LDL-R activity decreased significantly (p < 0.001) in Se deficiency. On 1 ppm selenium supplementation LDL-R activity increased significantly in comparison to adequate groups. On HCD feeding the receptor activity decreased significantly (p < 0.001) in all the three selenium status groups. After 2 months receptor activity decreased (p < 0.001) in Se deficient groups (Ia and Ib) and in HCD fed adequate group, whereas it increased significantly on 1 ppm selenium supplementation in comparison to 1 month treatment period (Fig. 4a &4b).

**Figure 5:**
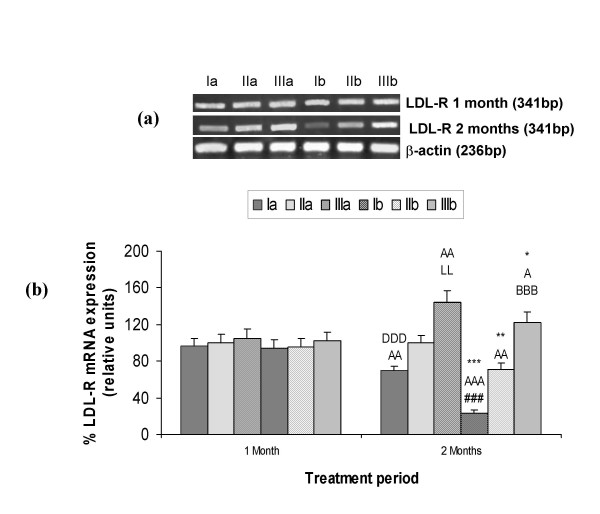
Hepatic LDL-R mRNA analysis in different groups: Ia-Se deficient control, IIa-Se adequate control, IIIa-Se excess control, Ib-Se deficient+HCD, IIb-Se adequate+HCD, IIIb-Se excess+HCD after 1 and 2 months. (a) mRNA expression by RT-PCR. β-actin was also amplified (house keeping gene). (b) bands were quantified by densitometric analysis. Data is expressed as mean ± SD from 4 observations. *p<0.05, **p<0.01, ***p<0.001 represent comparison between control and HCD groups; ^A^*p*<0.05, ^AA^*p*<0.01, ^AAA^*p*<0.001 comparison between 1 and 2 months; ^DDD^*p*<0.001 comparison between group Ia and IIa; ^###^*p*<0.001 comparison between group Ib and IIb; ^LL^*p*<0.01, comparison between group IIa and IIIa; ^BBB^*p*<0.001 comparison between group IIb and IIIb.

RT-PCR products of expected size i.e. 341 bp and 236 bp were obtained for LDL-R and β-actin. mRNA expression followed the same trend as it was observed for LDL-R activity i.e. no significant change was observed in expression after 1 month of treatment period. But after 2 months of diet feeding schedule mRNA expression decreased significantly (p < 0.001) in Se deficiency (Ia and Ib) in comparison to adequate groups i.e. 31% and 68% decrease was observed in groups Ia and Ib in comparison to IIa and IIb respectively. On 1 ppm Se supplementation, significant increase (p < 0.001) in RNA expression was observed in comparison to adequate groups. On HCD feeding in all the three selenium status groups significant decrease in the expression was observed. In Se deficient groups (Ia and Ib) and in HCD fed adequate group mRNA expression decreased and it increased significantly in 1 ppm Se supplemented groups after 2 months in comparison to 1 month data (Fig. 5a &5b).

### Apolipoprotein B levels

After one month of treatment, no significant change was observed in apoB levels by ELISA (Table 2). However after 2 months apoB levels in liver increased significantly (p < 0.001) in Se deficient control and high cholesterol diet (HCD) fed groups (Ia and Ib) in comparison to respective adequate groups (IIa and IIb). Whereas the level significantly (p < 0.001) decreased on 1 ppm Se supplementation in groups IIIa and IIIb. On HCD feeding a significant increase (p < 0.001) in apoB level was observed in comparison to respective control groups in all the three Se status groups after 2 months of diet feeding schedule. In selenium deficient groups (Ia and Ib), and in selenium adequate HCD fed group (IIb), the level increased and in 1 ppm selenium fed groups (IIIa and IIIb) it decreased significantly after 2 months of respective diet feeding in comparison to 1-month treatment period (Table 2).

**Figure 6:**
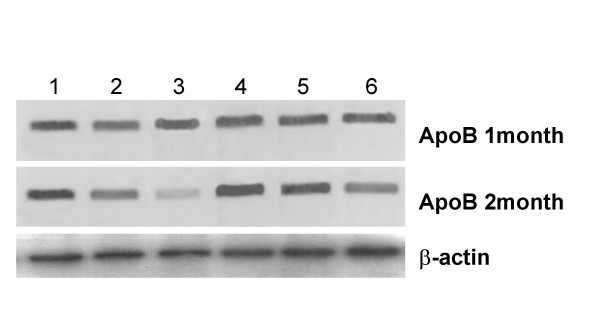
Western Blot analysis of apolipoprotein B. Protein samples (30 μg) were resolved on 7.5% SDS-polyacrylamide electrophoresis and then electrophoretically transferred to PVDF membrane. Membrane was probed with antibody against apolipoprotein B. Lane1-Se deficient control; Lane2-Se adequate control; Lane3-Se excess control; Lane4-Se deficient+HCD; Lane5-Se adequate+HCD; Lane 6-Se excess+HCD. β-actin was used as an internal control.

Apolipoprotein B expression by western blot followed the similar trend as it was observed in ELISA. After one month of treatment, no significant change in band intensity was observed. So no change was there in apoB expression on high cholesterol diet feeding as well as in selenium deficiency and on 1 ppm selenium supplementation (Fig. 6).

After 2 months, immunoblot band intensity was apparently higher in Se deficient control and HCD fed groups (Ia and Ib) in comparison to respective adequate diet fed groups (IIa and IIb). Whereas the intensity was lower in 1 ppm Se supplemented groups IIIa and IIIb in comparison to adequate groups IIa and IIb respectively. On cholesterol supplemented diet feeding, apoB protein expression was found to be increased in comparison to respective control groups in all the three Se status groups after 2 months. In selenium deficient groups (Ia and Ib), and in selenium adequate cholesterol fed group (IIb), apoB expression increased and in 1 ppm selenium fed groups (IIIa and IIIb), it decreased after 2 months of respective diet feeding in comparison to 1-month treatment period (Fig. 6)

## Discussion

Various studies suggested the prospective role of selenium in cardiovascular disorders [18]. Our results demonstrate that in selenium deficient animals significant increase in total cholesterol and LDL-cholesterol level was observed in comparison to adequate selenium fed animals [19]. Increased LDL is accumulatedin the intima, where it is oxidized. This oxidized LDL is associated with increased risk of coronary heart disease [20]. On 1 ppm Se supplementation lipid levels decreased significantly [21], Se supplementation might be protecting the LDL from oxidative modifications and further atherogenic changes [22,23]. Further, selenium supplementation leads to an increase in HDL cholesterol fraction [18]. HDL fraction increases the cholesterol elimination from tissues including smooth muscle cells in the aorta wall and facilitate the cholesterol transport to the liver, thus preventing its deposition and formation of atheromatous plaque [24].

In the present results in Se deficient groups, hepatic glutathione peroxidase (GSH-P_x_) activity decreased significantly in comparison to adequate groups (Table 1), so these observations confirm the Se deficiency, which is associated with decreased GSH-Px levels [25]. On high cholesterol diet feeding GSH-Px activity increased in all the groups. This increase in Se dependent GSH-Px on HCD feeding is attributed to the increased lipoperoxidative stress associated with cholesterol feeding as previously established in our lab [21]. Animals fed with the Se deficient diet had decreased hepatic 5'-DI activity as well as mRNA expression in comparison to the Se adequate groups [16,17]. During selenium deficiency hepatic stores of Se might be insufficient to allow the synthesis of 5'-DI. On 1 ppm Se supplementation GSH-Px and 5'-DI levels increased in control as well as HCD fed groups, this could be due to the fact that being selenoproteins the level of these two enzymes increased on Se supplementation.

Decreased level of T_3 _in selenium deficiency as well as on HCD feeding might be owing to the decreased conversion of T_4 _to T_3 _in the liver and other parts due to decreased 5'-DI (selenoprotein) expression during Se depletion [17]. Present studies revealed that in Se deficiency the LDL-R activity as well as mRNA expression is down regulated in comparison to adequate Se diet fed animals after 2 months of diet feeding (Fig. 3 &4). This could be due to the decreased T_3 _level during Se deficiency. T_3 _is directly involved in the regulation of LDL-R expression via modulation of SREBP-2 (sterol regulatory element-binding protein-2) gene expression. SREBP-2 is a major transcriptional regulator of cholesterol uptake through LDL-R [26]. In the present study we have observed that in Se deficiency, cholesterol level increased, this increased intracellular cholesterol level might be another reason to down regulate the LDL-R expression through feedback signaling pathway [27]. On feeding high cholesterol diet to the animals, LDL-R activity and mRNA expression decreased in all the three selenium status groups, so exogenous cholesterol given through diet is being used in the signaling pathway and probably it is suppressing the transcription of LDL-R through feedback mechanism. In case of 1 ppm Se supplemented groups increased T_3 _level might be upregulating the receptor expression.

In the current studies, on high cholesterol diet feeding for 2 months, apoB expression increased in all the three groups (Table 2; Fig. 6). This increased level of apoB on high cholesterol feeding is due to decreased expression of LDL-R during hypercholesterolemia as observed in the present studies. Decreased level of LDL-R is responsible for decreased clearance of apoB along with LDL, so these apolipoproteins are accumulated in the body [28,29]. During Se deficiency apoB levels in liver increased significantly in the present studies [30]. This could be due to the reason that selenium deficiency leads to decreased T_3 _levels and inturn hypothyroid state through decreased expression of 5'-DI enzyme [17,31], further hypothyroidism has been associated with increased level of apoB [14]. As T_3 _is involved in LDL-R expression, so reduced T_3 _levels during selenium deficiency is responsible for increase in apoB levels. Staels et al. [32] demonstrated that thyroid hormones activate the LDL-R, leading to an increased fractional catabolic rate of apoB without influencing its synthesis rate. Davidson et al. [33] reported that T_3 _administered to hypothyroid animals reduced the plasma apoB concentrations.

In the present studies on 1 ppm selenium supplementation for 2 months decreased expression of apoB was observed (Table 2; Fig. 6). This could be due to the increased level of T_3 _observed in the present study on Se supplementation through increased 5'-DI expression. So it results in increased catabolic rate of apoB through increased LDL-R expression. Davidson et al. [34] reported the suppressed synthesis of apoB during T_3 _supplementation. Walton et al. [35] suggested the increased catabolism of apoB through LDL receptors during hyperthyroidism. It is probably a combination of suppressed apoB synthesis and its increased elimination via LDL receptors during selenium supplementation here, which is regulating the apoB expression through T_3 _levels.

After one month of treatment, no significant change in the LDL-R and apoB expression was observed, this might be due to the reason that after 1 month the cholesterol accumulation might not be up to the extent that it could stimulate the feedback signaling pathway at translational as well as at transcriptional level to regulate LDL-R expression. So apoB catabolism through LDL receptors was not affected in different groups.

## Conclusion

So, these results form the basis for a model that selenium status in the body regulates apolipoprotein B expression through selenoenzyme, 5'-DI. Selenium deficiency leads to decreased expression of 5'-DI, decreased T_3 _levels and decrease in LDL-cholesterol removal from blood through downregulation of LDL-R mRNA expression, ultimately decreased apoB catabolism through LDL receptors. Whereas Se supplementation upto 1 ppm leads to decreased apoB expression through increase in the LDL-R mRNA expression again via modulation of 5' -DI expression and in turn has the protective role against hypercholesterolemia. However, this interrelationship between selenium status and apoB expression warrants further investigation to decide the precise mechanism of lipid metabolism through the effect of Se status on the apolipoprotein B expression. Further studies must be undertaken to explore the therapeutic role of selenium supplementation in hypercholesterolemia.

## Methods

### Experimental Animals

Young male Sprauge-Dawley rats (100 g-body weight) were used in the present study. Animals were obtained from the Central Animal House, Panjab University, Chandigarh.

### Treatment Protocol

Animals were acclimatized to the laboratory animal room and divided into three groups initially, group I (Se deficient diet fed), group II (Se adequate diet fed) and group III (Se excess diet fed). Feed and water were given *ad libitum*. This Se diet was given to the animals initially for 10 days so as to achieve the required Se status. The animals in these three groups were further divided into two each viz.: Group Ia (Se deficient control), Group Ib (Se deficient + high cholesterol diet fed); Group IIa (Se adequate control), Group IIb (Se adequate + high cholesterol diet fed); Group IIIa (Se excess control), Group IIIb (Se excess + high cholesterol diet fed). Treatment protocol was for 1 and 2 months.

### Diet preparation

Yeast based synthetic Se deficient diet (supposed to contain 0.02 ppm Se) was prepared in the laboratory itself according to the composition given by Burk [36]. It contained torula yeast (inactivated) 30%, sucrose 56.99%, corn oil 6.67%, mineral mix 5%, vitamin mix 1%, dl-methionine 0.3% and vitamin E 0.04%. Se adequate and excess diet was prepared from Se deficient diet by supplementing it with 0.2 ppm and 1 ppm of Se as sodium selenite (Sigma Chemicals). 2% of cholesterol (Loba-Chemie, India) was added to the respective high cholesterol diet (HCD) groups.

After completion of diet feeding schedule, rats were kept on fasting for 10 hrs, anesthetized and exsanguinated. Serum and tissue (liver) samples were collected from each animal. Tissues were snap frozen in liquid nitrogen. Serum total cholesterol and LDL-cholesterol levels were estimated by enzymatic colorimetric kits obtained from E. MERCK diagnostic (Germany). Various parameters were carried out as detailed below

### Selenium estimation

Selenium level was estimated by fluorimetric method [37], based on the principle that Se content in serum or tissue on acid digestion is converted to selenous acid. The reaction between selenous acid and aromatic-o-diamines such as 2,3-diamino-naphthalene (DAN) leads to the formation of 4,5-benzopiazselenol, which displays brilliant lime-green fluorescence when excited at 366 nm in cyclohexane. Fluorescence emission in extracted cyclohexane was read on fluorescence spectrophotometer using 366 nm as excitation wavelength and 520 nm as emission wavelength.

### Se-dependent GSH-Px activity

Glutathione peroxidase (GSH-Px) activity was assayed using H_2_O_2 _as substrate [38]. The assay was carried out in the post-mitochondrial fraction (PMF) of liver as already published by us [39], the activity was expressed as μmoles of NADPH oxidized/min/mg protein. Total protein was done in all the samples [40].

### T_3 _and T_4 _levels

Serum T_3 _and T_4 _estimation was done by radioimmunoassay (RIA) kits procured from BARC, Mumbai (Cat. No. RIAK-4/4A and RIAK-5/5A for T_3 _and T_4 _respectively).

### Type-I 5'-iodothyronine deiodinase activity

Type-I iodothyronine deiodinase (5'-DI) activity in liver was estimated by following the method of Behne et al. [41].

### LDL-R activity

LDL-R activity was estimated *in vivo *by following the method of Brown and Goldstein [42] and as per methodology already used by us [43]. Briefly, LDL isolated from overnight fasting human plasma using a single vertical spin density gradient ultra centrifugation [44], was radiolabeled with Na [^131^I] using chloramine-T [45]. Separation of labeled protein and unreacted iodide was done by gel filtration through sephadex G-25 column. The sterilized radiolabeled LDL was injected to rats of different groups. One ml of blood was taken 2 hrs after injection to measure the counts of radiolabeled LDL, and considered as counts at zero time interval or initial counts. Subsequently counts were also taken at 24, 48, 72, 96 and 120 hrs after injection. Percent decrease in counts at increasing time interval was taken as a measure of clearance of LDL from animal blood and in turn as indirect measurement of the LDL-R activity.

### Apolipoprotein B levels by ELISA

Apolipoprotein B concentration was estimated in liver by ELISA using species specific (polyclonal anti rat apoB) antibody (Santa Cruz Biotechnology, Inc. Santa Cruz, CA). Optical density of each well was measured at 405 nm in ELISA reader (Stat Fax; Awareness Technology Inc., USA).

### mRNA analysis

The mRNA expression for 5'-DI andLDL-R was done in liver using RT-PCR kit from QIAGEN.

#### RNA isolation

Total RNA from liver was extracted using TRI REAGENT (Molecular Research Centre, Inc. Ohio). The integrity and size distribution (quality) of RNA was examined by formaldehyde agarose gel electrophoresis.

#### RT-PCR

2 μg of total RNA template from different groups after treatment with DNase I (Ambion) was used in RT-PCR reaction. To the reaction mixture added 10 μl of 5X QIAGEN OneStep RT-PCR buffer (2.5 mM MgCl_2 _as final concentration), 2 μl of dNTP mix (10 mM of each dNTP), 5 μl of each forward and reverse gene specific primers (from 10 μM stock), 2 μl QIAGEN One Step RT-PCR Enzyme Mix, 1 μl RNase inhibitor (1 U/μl) and finally 25 μl of PCR grade RNase-free water (provided in the kit) to make total volume 50 μl. Mixed it gently by vortex and centrifuged it to collect all the components at the bottom of the PCR tubes. The PCR reaction was performed in the thermal cycler (Techne Ltd. England) using following conditions: the RT reaction was performed at 50°C for 50 min, initial PCR activation was done at 95°C for 15 min, followed by 35cycles of 94°C (denaturation) for 45 sec, 58.8°C (annealing) for 45 sec and 72°C (extension) for 1 min. Finally, incubated at 72°C for 10 min to extend any incomplete single strands.

Optimal oligonucleotide primer pairs for RT-PCR were selected with the aid of the software Gene Runner. The primer sequence (5' to 3') for rat 5'-DI gene coding (+) strand was TCTGGGATTTCATTCAAGGC, noncoding strand was TAGAGCCTCTCAGGCAGAGC, LDL-R gene coding (+) strand was ACCGCCATGAGGTACGTAAG, noncoding (-) strand was GGGTCTGGACCCTTTCTCTC and for rat β-actin gene coding (+) strand was AGAGCTATGAGCTGCCTGAC, and the noncoding (-) strand was CTGCATCCTGTCAGCCTACG. The length of RT-PCR products for 5'-DI, LDL-R and β-actin were 346, 341 bp and 236 bp respectively.

Final PCR products were analyzed on 1.5% agarose gel electrophoresis using 10 mM TE buffer. 5 μl of PCR product was used from each tube. Densitometric analysis of the bands was done by UviBandMap software (Uvitech, England).

### Western Immunoblot Analysis

Western immunoblot analysis for apoB was done in liver. Tissue homogenate (10% w/v) from each group prepared in 20 mM tris-HCl buffer (pH 7.4) at 4°C was centrifuged at 3000 g for 10 min and the supernatant was used in the assay. Protein sample (30 μg) from each treatment group was separated by 7.5% SDS-polyacrylamide gel electrophoresis using minigel apparatus (BIORAD, UK). The separated proteins were electrophoretically transferred to PVDF membrane (Immobilon-P, Millipore, USA). Membrane was probed with antibody against apoB (Santa Cruz Biotechnology Inc. Santa Cruz, CA). β-actin was used as an internal control. Immunoblot analysis for β-actin was also done by using antibody against β-actin (Oncogene, USA).

### Statistical analysis

Data is expressed as mean ± SD. Difference between different groups was tested using student's t test for unpaired values.

## Authors' contributions

Both the authors SD and MPB have made substantive contributions to this study. Each author has participated in the work to take public responsibility for appropriate portions of the content. Both the authors have read and approved the final manuscript
